# Single Stock Dynamics on High-Frequency Data: From a Compressed Coding Perspective

**DOI:** 10.1371/journal.pone.0085018

**Published:** 2014-02-21

**Authors:** Hsieh Fushing, Shu-Chun Chen, Chii-Ruey Hwang

**Affiliations:** 1 University of California Davis, Davis, California, United States of America; 2 Institute of Mathematics, Academia Sinica, Taipei, Taiwan; Universidad Veracruzana, Mexico

## Abstract

High-frequency return, trading volume and transaction number are digitally coded via a nonparametric computing algorithm, called hierarchical factor segmentation (HFS), and then are coupled together to reveal a single stock dynamics without global state-space structural assumptions. The base-8 digital coding sequence, which is capable of revealing contrasting aggregation against sparsity of extreme events, is further compressed into a shortened sequence of state transitions. This compressed digital code sequence vividly demonstrates that the aggregation of large absolute returns is the primary driving force for stimulating both the aggregations of large trading volumes and transaction numbers. The state of system-wise synchrony is manifested with very frequent recurrence in the stock dynamics. And this data-driven dynamic mechanism is seen to correspondingly vary as the global market transiting in and out of contraction-expansion cycles. These results not only elaborate the stock dynamics of interest to a fuller extent, but also contradict some classical theories in finance. Overall this version of stock dynamics is potentially more coherent and realistic, especially when the current financial market is increasingly powered by high-frequency trading via computer algorithms, rather than by individual investors.

## Introduction

One Wall Street adage on asset returns says that “it takes volume to move the price.”

Underlying this adage is the classic theory which claims that return volatility is generated from trading volume (see [1], and, for review [2], [3] and the references therein). Later, this theory is modified to assert that return volatility is indeed generated from transaction number (see [4], and [5]). The supporting evidence is based on a positive correlation between absolute price changes and transaction number, and the absence of a significant correlation increment by conditionally including trading volume. Ane and Geman (2000) demonstrates further evidence supporting this modification by showing that the normality of asset returns will be revealed only along the transaction clock.

Specifically, consider the dynamic linear regression setup used in [5]. Denote return, trading volume and transaction number of one single stock at time 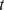 by a triplet 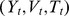, with 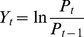 computed from the stock price 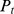. The absolute price change, also called the 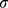-volatility, is computed in the following fashion:
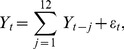
(1)while an estimate of the 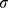-volatility at time 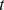 is computed as:



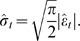
(2)Then the employed dynamic linear regression model with 12-lags is:
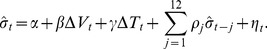
(3)


The reported 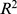 values for this regression model on all data analyzed in [5] are less than 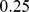. Therefore, a natural question arises: Can both theories be trustworthy while missing at least 75% of the total sum-of-squares in a goodness-of-fit?

**Figure 1:**
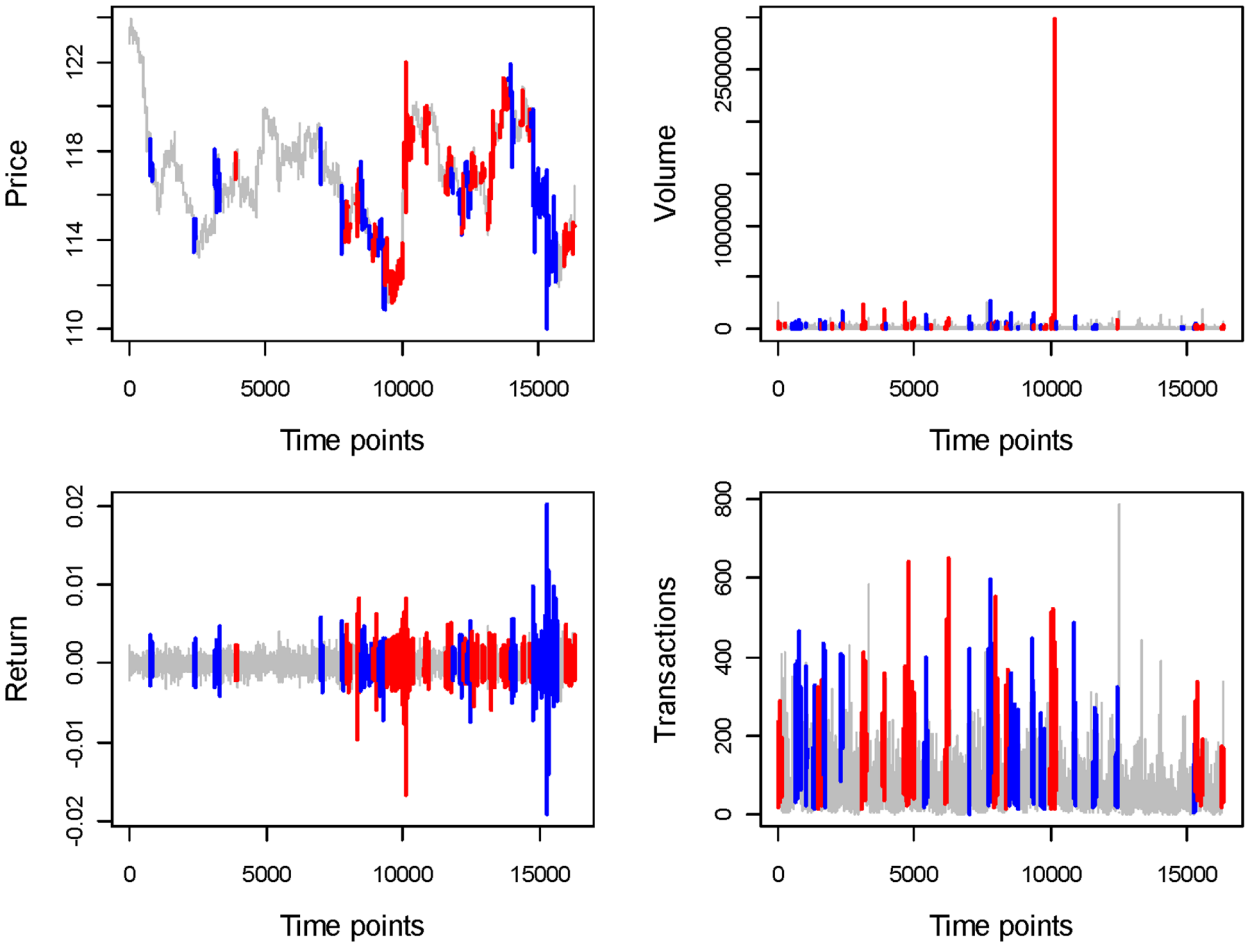
Three dimensions of IBM stock with volatility marked with color. The red marking denotes a positive price change between the onset and offset of a marked segment, while the blue marking denotes a negative price change.

This low 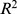 value indicates that the above local linear regression setup may have missed some very important perspectives about the stock dynamics under study. It further implies that, without thorough data exploration to gain as much empirical understanding as possible about the stock dynamics, direct modeling may have difficulty capturing the intricate dynamic structures embedded within high-frequency data. To a great degree this fact can be envisioned by looking at the three-dimensional time series of a single stock, as shown in Figure 1 below. These series are full of versatile micro-structures that have proven to be too difficult to model simultaneously. Hence the question facing us now is: how we can properly explore a single stock’s dynamics to obtain its intrinsic dynamic patterns as a basis for modeling? This is the question that motivates this study.

As an analogy of the task facing us here, the stock dynamics are like a teeming cauldron of phenomena present in the return, trading volume and transaction number series and waiting for exploration, classification and analysis. We attempt to snatch out of this cauldron some specific things which lend themselves to a precise analysis. These things must preserve the essence of the dynamic aspects of the stock’s complexity.

The complexity of high-frequency data mandates that the original data be compressed into digital code sequences for easy exploration. Ideally, this compression needs to achieve two aspects of data summary. On one hand, the compressed version needs to preserve the important dynamic signatures belonging to each individual dimension of a stock. On the other hand, the coupled version of the resultant code sequence must be simple enough to make dynamic pattern recognition feasible and manageable.

To achieve this data compression goal we propose the following resolution: It is postulated that a latent binary state-space trajectory governs the transitions of each high-frequency time series in and out of its pertinent equilibrium state. Here the equilibrium state is heuristically taken to be a temporal segment containing a sparsity of some extreme event, while in contrast the off-equilibrium state contains an aggregation of the extreme event. The large absolute return is a natural choice for an extreme event on the return dimension. This extreme event choice is employed primarily due to the scale-invariance property of its recurrence time process. The empirical evidence of such scale-invariance was reported in Fushing et al. (2010), while the theoretical justifications are recently established in [6], [7].

For expositional simplicity, we term the off-equilibrium state on the return series as volatility, while the equilibrium state is termed non-volatility. We encode each state as 1 and 0, respectively. Similarly, large values for trading volume and transaction number are natural choices for extreme events for the other two dimensions. In the same spirit of aggregation versus sparsity of extreme events, volatility versus non-volatility is designated for the two contrasting states on the dimensions of trading volume and transaction number as well. Therefore, volatility is unified to denote the manifestation of the collective effects of trading strategies exerted on the three dimensions–return, trading volume and transaction number–of a single stock. Specifically speaking, the state of return volatility indicates the persistent heterogeneity in perception regarding to a stock’s price among traders. The trading volume volatility indicates the persistent occurrences of large amounts of shares being traded. And the trading number volatility indicates the persistent trading activities. Thus the notion of volatility here bears the pertinent dynamic meaning and no longer stands for a univariate quantity of absolute price changes, as calculated via 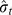.

Throughout this paper we do not assume a priori knowledge about the stochastic mechanism governing the two states’ transitions, such as a Markovian structure. Without such assumptions, any likelihood-based statistical inference, such as the maximum likelihood approach, is muted. Instead, we rely on the computational technique for segmenting the extreme event’s pattern of aggregation versus sparsity along each dimension of the time series. Specifically, we decode the volatility and non-volatility states by using a nonparametric computing algorithm, called hierarchical factor segmentation (HFS). This algorithm is detailed under a finance context in [8], while its original development with regard to animal behavior is given in [9], and a wide-range of successful applications are presented in [8], [10], [11], [12], [13], [14]. This HFS algorithm is independently applied to each of the three dimensions to result in three 0–1 time series. These three binary time series are coupled together, so that there are 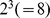 combinatorial states computed along the discrete time axis pertaining to the original 30 second temporal resolution of high-frequency data. In this fashion we estimate the latent state-space trajectory underlying the original single stock’s dynamic data with a base-8 coding sequence of the same length.

We then compress the digital code sequence by simply recording its sequence of transitions among the 8 states. That is, by taking away the duration information of each state, the compressed digital code sequence becomes much shorter than the estimated digital sequence of the state-space trajectory. Through this compressed digital sequence we can easily extract information about which dimension’s volatility is leading the others, so that it yields precise information regarding the roles of trading volume and transaction number in the return’s volatility formation. In summary, the chief merit of our computational approach using such a compressed digital sequence is the capacity to manifest the causal relationship among the three dimensions of stock dynamics. In sharp contrast, the linear trend brought out by regression analysis based on equation (3) is unidirectional. Hence it should not bear the causal implication carried by the classic theory or the Wall Street adage. We also indicate why the linear model equation (3) produces such a low 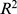 value.

All coding algorithms, estimation of the state-space trajectory, and the compression scheme are discussed and illustrated through IBM stock from the year 2005 to the year 2009 in Section 2. We evaluate the credibility of all computed dynamic patterns by incorporating the information about state duration in Section 3. By using the codewords’ frequencies and their proportion of overlap among the three dimensions’ volatility states, we conclude that our causal patterns are very meaningful and credible. At the end we also investigate how our computed dynamic patterns vary when the global stock market transitions into and out of contraction and expansion cycles in Section 4. A collection of related issues are briefly discussed in the last section.

## Methods

Suppose that there is a binary state-space trajectory 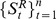 underlying the return time series 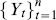 of length 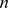, while 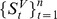 underlies the trading volume time series 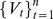 and 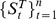 underlies the transaction number time series 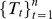. All state-space variables 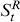, 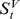 and 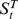 take the value 0 when in the non-volatility state and the value 1 when in the volatility state. Here the volatility state attempts to capture the extreme events’ aggregation pattern. In contrast, its immediate neighboring segments are marked by non-volatility for the sparsity pattern. Hence the task of estimating a binary state-space trajectory is equivalent to performing pattern recognition by segmenting aggregation versus sparsity for a chosen extreme event.

Here we employ a segmentation algorithm without a priori knowledge about the stochastic mechanism of transitions between the 0 and 1 states. This non-parametric computational approach is called the hierarchical factor segmentation (HFS) algorithm. The construction and applications of the HFS algorithm to high-frequency asset price data are detailed in [8]. This algorithm is applied independently on each dimension to respectively decode 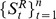, 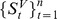 and 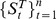. The three individual segmentations are illustrated in Figure 1 with color-marked segments for volatility.

The computed segments of non-volatility and volatility states indeed bring out a very characteristic difference in stock dynamics. As reported in [8], the return’s non-volatility state is characterized by an equilibrium of transitions between positive and negative returns, while the volatility state is characterized by an off-equilibrium that is biased toward either the positive or negative returns, depending on the price differences. Furthermore, from the distributional aspect, the distribution of returns in the non-volatility state are more concentrated around zero than that of returns in the volatility state. Their standard deviation ratio is nearly 1 to 2. From this perspective, we see that there is indeed a segment-by-segment mixture within stock dynamics, but not the point-by-point mixture as used in [1]. More descriptive and dynamic differences are reported in [8].

As far as exploring stock dynamics is concerned, we focus only on the volatility state in this paper. The reason for this is that, from a conceptual driving-force perspective, when there is a manifestation of volatility occurring on any one of the three dimensions at any time point, it implies that large and small market participants are collectively exerting their influence upon the stock dynamics of interest through their trading strategies. Therefore, a volatility state seems like an open window for peeking into the stock dynamics. Specifically, we look for overlapping open windows on different dimensions. When they overlap, they are connected due to their manifestation of the same driving force. Their distinct onset time points signal the causal aspects of the driving force on the stock dynamics, in a fashion of one leading the other, or vise versa. Furthermore, all causal types of association relationships are represented in terms of codewords, as discussed in the next subsection. Hence the proper association measurement can be easily evaluated through the frequency. This paradigm explains the chief merit and advantages of taking a compressed coding approach for exploring stock dynamics.

### Digital Coding via the HFS Algorithm and its Compression

After applying the HFS algorithm independently and individually on the return, volume and trading number time series, we encode the original high-frequency data into a digital code sequence using the following coding scheme.

We embed 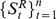, 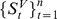 and 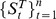 on the same discrete temporal axis from 1 through 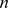. At each time point, one of the 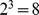 combinatorial states of the three dimensions, say 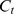, is assigned using the formula:
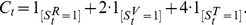
(4)


Hence code 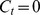 indicates the simultaneous occurrence of non-volatility states for the three dimensions. In contrast, code 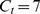 indicates very distinctively the “synchrony” of volatility. Code 1 indicates only volatility in the return; while code 6 is for simultaneous volume and trading number volatilities, but lacks volatility in the returns. Codes 3 and 5 indicate volatility of the returns coupled with that of trading volume and transaction number, respectively; codes 2 and 4 indicate individual volatility of volume and trading number, respectively.

**Figure 2:**
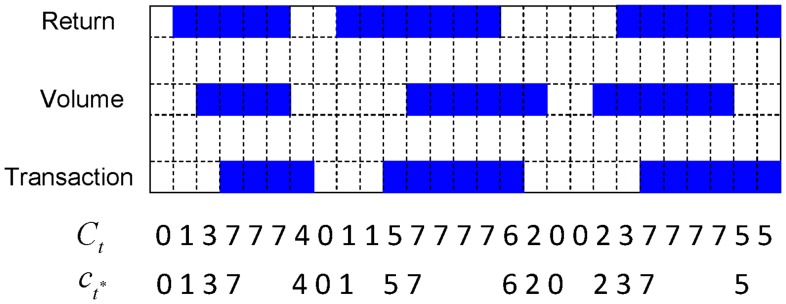
Schematic coding illustration.

On the computed digital sequence 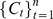, each distinct code, 0 through 7, typically replicates itself before transiting to a different code, as illustrated in Figure 2. Thus we can further compress 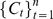 into a much shorter state transition sequence, denoted by 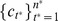, by deleting the repeated codes, that is, by removing all the duration information from 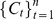. An illustrated example of 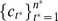 is given in the bottom panel of Figure 2. Here 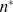 becomes much smaller than 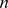. A non-zero code segment on 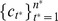 typically means a code segment between two successive 0 codes, such as code segment 15762 in Figure 2.

**Figure 3:**
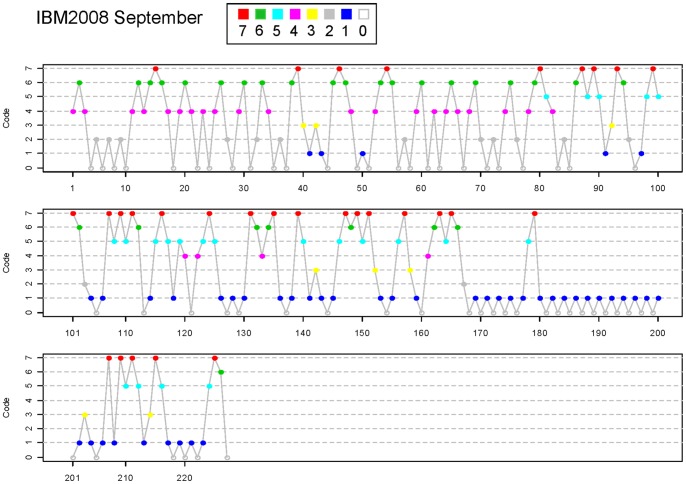
A digital representation of IBM stock in the month of September 2008.

A digital code sequence 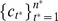 computed from real data is given in Figure 3. This code sequence gives a very concise summary of what this stock has been through in the midst of the recent recession. From the 10th code onward in the upper panel, the code segment 4646764 states that the transaction number’s volatility with code 4 leads and overlaps the trading volume’s volatility to become code 6. Then the trading volume’s volatility is lost, leaving the transaction number’s volatility alone. The persistent transaction number’s volatility again leads and overlaps with the trading volume’s volatility to become code 6, and subsequently the return’s volatility is generated to enter a state of synchrony of volatility. However the return’s volatility does not last, so the trading volume’s disappears. Finally, the persistence of the transaction number’s volatility also disappears as the stock’s dynamics fall into the equilibrium. It is clear that the driving force manifested through the transaction number’s volatility was very persistent and strong during the time period giving rise to this code segment. However, we do not need to distinguish whether the driving force indeed primarily comes from a strategic model or a completive model with multiple informed traders, or whether both are the chief mechanisms underlying this stock’s dynamics.

We then see several code segments of 464, 264 or 462. These are unsuccessful attempts at stimulating the return’s volatility. From the 77th code onward to the end, the dynamic patterns on the code sequence manifest very distinct changes. More of the return’s volatility is present, so there are more code segments like 137, 157, and even 17. These dynamic patterns jointly point out that it is more likely for the return’s volatility to stimulate the volatility of both the transaction number and the trading volume to achieve synchrony, than the other way around. In the middle panel, we see two subsequences of code, 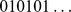. This kind of subsequence of code is not rare, that is, it is not uncommon to see the presence of the return’s volatility unaccompanied by the other two volatilities. It is very likely that this phenomenon contributes significantly to the fact that the 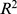 value of the linear regression model (3) is less than 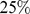. A collection of 60 monthly 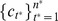 code sequences on IBM stock from 2005 to 2009 is established for analysis purpose of this study.

### Computed Dynamic Patterns

**Figure 4:**
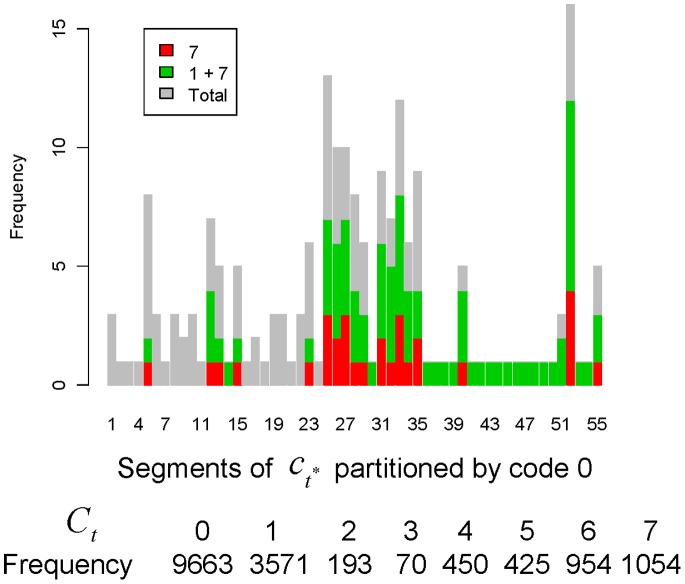
Prevalence of code 7 and code 1 and 7 in a code sequence of IBM stock, September 2008. Composition of codes 1 and 7 within the code-segment bar partitioned 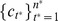 by all codes 0. The bottom row records the total duration lengths for each of the 8 codes on 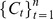.

Before discussing the interesting and important dynamic patterns, we give an overview of the composition of code segments on the code sequence 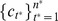. The bar plot of Figure 4 reveals that code segments with length larger than 3, that is, that involve at least 3 non-zero code transitions, contain codes 1 and 7. This observation points to two patterns. First, synchrony code 7 and return’s volatility code 1 are highly associated within this particular month. Second, a code segment that does not contain codes 1 or 7 does not last long, that is, the volatilities of trading volume and transaction number are likely to be failed attempts at stimulating return’s volatility. In contrast, the single code 1 may have been undetected. The bottom panel of Figure 4 shows that synchrony (code 7) is prevalent throughout the code sequence 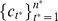 as well as 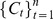 when the duration length is considered. It has nearly half the prevalence of the volatility of trading volume, or of transaction number. Interestingly, codes 1 and 7 outnumber codes 2 and 4 in terms of the numbers of code transitions and the code duration length.

These observed patterns are nearly universal among the 60 months of IBM stock from 2005 to 2009. Putting together these observations, we can conclude that neither the volatility of trading volume nor that of transaction number is likely to take the leading role in stimulating the return’s volatility.

**Figure 5:**
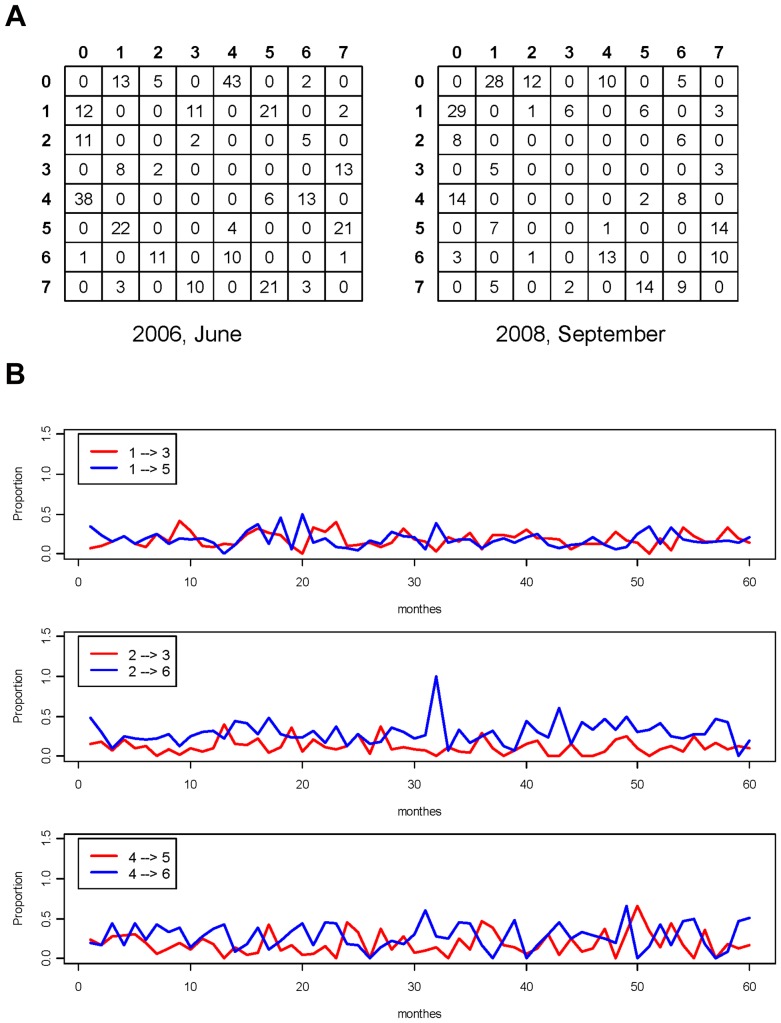
Code transition matrices and monthly proportions. (a) Upper (matrix) panel: Two transition matrices for compressed digital code sequences computed based on monthly IBM stock for June 2006 and September 2008, with a 30 sec. sampling rate; (b)Lower (time series)panel: Comparing monthly proportions of order-2 codewords from 2005 through 2009∶13, 15, 23, 26, 45, 46.

On the contrary, the return’s volatility may have taken the leading role to stimulate the other two volatilities, and to frequently arrive at synchrony of the stock’s dynamics. Specifically, based on the compressed digital code sequence 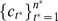, we can compute the prevalence of the dynamic pattern that can be expressed in terms of codewords of various order, such as 13 or 137. The order-2 codeword 13 means that the return’s volatility leads and overlaps with that of the trading volume, and codeword 137 means that the dynamic pattern 13 propagates synchrony of the stock dynamics. First, the prevalence of the collection of order-2 codewords can be easily evaluated and summarized into a code-to-code transition matrix, as illustrated in Fig. 5(a).

Beyond indicating the transition probability, it is emphasized once again that the proportion of code transitions in each row is also causally informative. For instance, in the panel for June 2006, we see the following: On one hand, the return’s volatility (code 1) is more likely to stimulate the transaction number’s volatility (code 4) into codeword 15, with a rate of 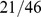, than to stimulate the trading volume’s volatility (code 2) into codeword 13, with a rate of 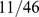. On the other hand, the volatilities of trading volume and transaction number are more likely to stimulate each other into codewords 26 and 46, respectively, than to lead the return’s volatility into codewords 23 or 45. Similar dynamic patterns are also seen in the panel for September 2008. These patterns are not universal among all 60 monthly analyses on IBM stock from 2005 through 2009. The summarized ratios for six order-2 codewords are given in Figure 5(b). These dynamic patterns together provide considerable strong evidence to contradict the Wall Street adage and the corresponding theories in finance.

**Figure 6:**
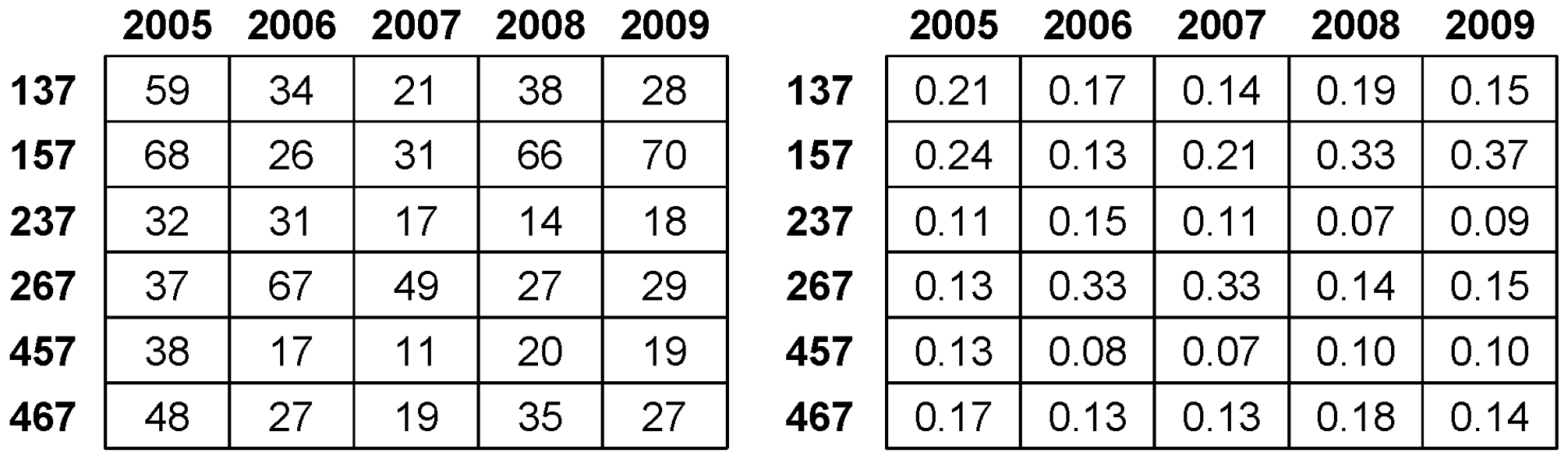
Relative yearly prevalence of order-3 code segments ending with code 7 from years 2005 to 2009. Table on the left reports yearly frequencies, while table on the right reports the relative frequencies among the six code segments.

Additional important dynamic pattern information is seen throughout the five-year analysis of order-3 codewords leading to code 7, as reported in Figure 6. Both tables reveal that the return’s volatility constantly takes the role of leading the volatilities of the other two dimensions toward synchrony throughout the five years of IBM stock dynamics. Specifically, the sum of the relative frequencies of codewords 137 and 157 is larger than 50% for years 2008 and 2009. Further interesting observations are also visible. First, by comparing the sum of the relative frequencies of codeword 237 and 267 with that of codewords 457 and 467, we see that the trading volume’s volatility has at least as much potential to generate synchrony as the transaction number’s volatility. Second, by comparing the sum of the relative frequencies of codewords 237 and 457 (less than 25%) with that of codewords 267 and 467 (larger than 29%), we conclude that the trading volume’s volatility and the transaction number’s volatility are likely to stimulate each other, and then to together stimulate the return’s volatility.

By summarizing all the aforementioned observations, we can conclude that the old Wall street adage and the classical theories about taking the trading volume or the transaction number to move the asset return or price are true only for a very limited percentage. For the major percentage, the stock dynamics operate in the reverse direction: It takes the return to stimulate the volatilities of the trading volume and transaction number. Then they both prolong the return’s volatility. This conclusion is more in line with another Wall Street adage among traders: “(return’s) volatility is equal to profit.” This revised version of finance “theory” might be even more coherent with the current high-frequency trading performed by computer algorithms.

**Figure 7:**
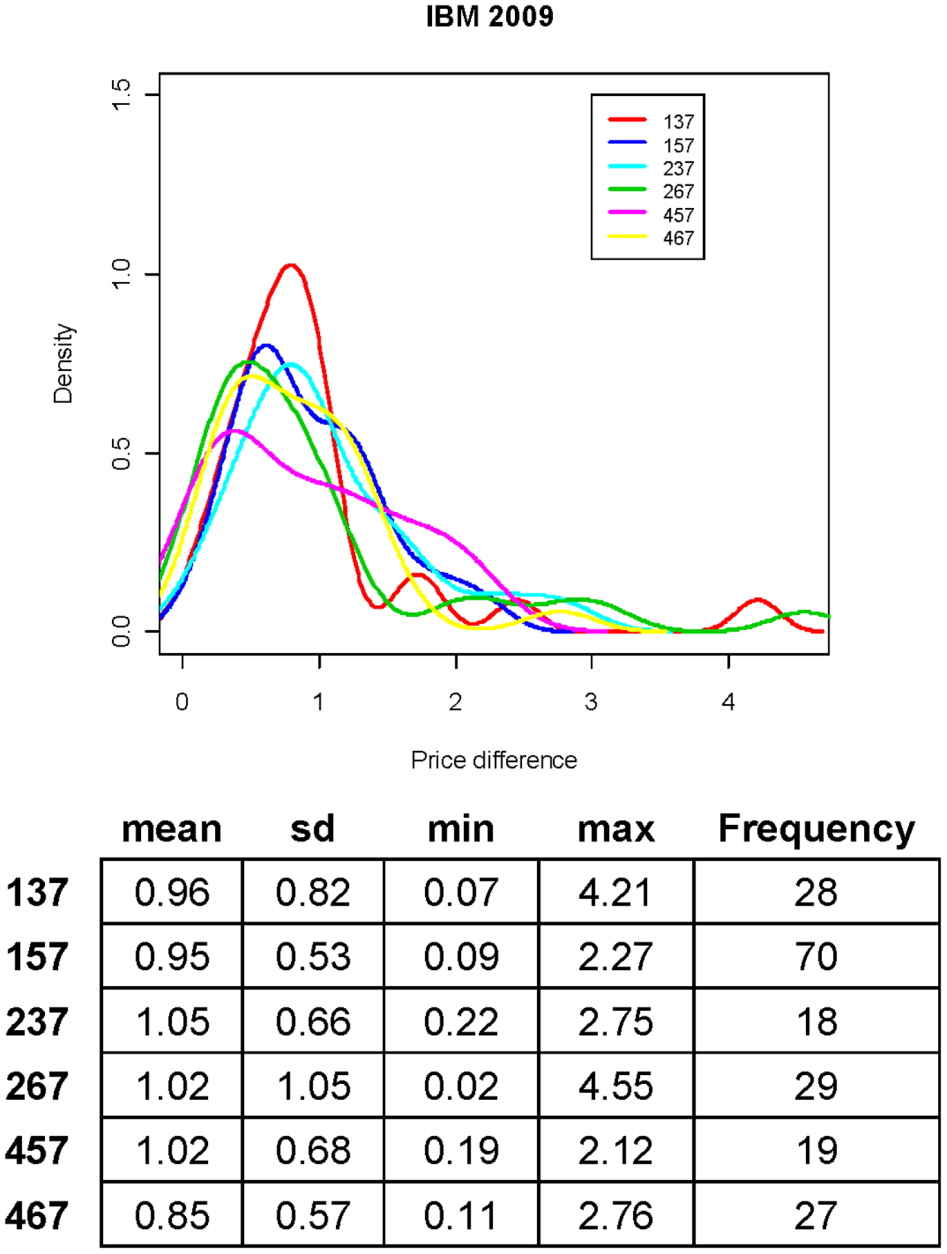
The distribution of price difference (segment: 7) of triple codes: 137, 157, 237, 267, 457 and 467.

At the end of this section, we also report two complimentary pattern information of stock’s dynamics: One is about overall price changes between on-set and off-set of a synchrony; the other is about the rate of synchrony deformation. Six scenarios of synchrony are considered in Figure 7. Six kernel smoothed densities of absolute changes between prices at onset and offset of code-7 segments are reported in the upper panel of Fig. 7, while the corresponding distributional characteristics are reported in the lower panel of Fig. 7. The common characteristic feature of the six densities is its extended long tail. This long tail phenomenon indicates that code-7 captures many instances of large up or down movements of stock prices.

**Figure 8:**
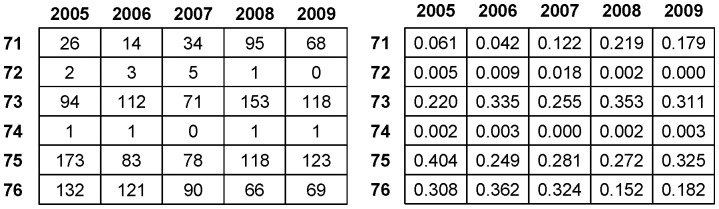
The frequency table of codewords after achieving code 7.

Furthermore, in Figure 8, we see that the dynamic pattern of synchrony deformation is primarily due to loss of the volatilities of trading volume and transaction number. The sum of the percentages for codewords 71, 73 and 75 is always more than 60%. It goes as high as 80%, especially during the two consecutive years 2008 and 2009. Both years are during the computer trading era at the New York Stock Exchange. This dynamic pattern implies that trading behavior has undergone a drastic change, and has consequently affected the stock dynamics significantly.

## Analysis

In this section we develop a new way of evaluating the causal association for a binary time series. This version can be easily extended into a multivariate setting. It is understood that an order-2 codeword based on 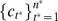, like any one of those considered in the previous section, corresponds to an overlap of two HFS-computed segments on two different dimensions of the stock dynamics. For instance, the codeword 13 based on 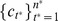 means that the return’s volatility segment overlaps with the trading volume’s volatility segment. Likewise for codeword 31. By collecting all the overlapping subsegments of two kinds of volatilities, we may compute the relative proportion of one subsegment’s overlap with respect to the total length of either one of the volatility segments. These overlap proportions are correspondingly estimating the 6 conditional probabilities:
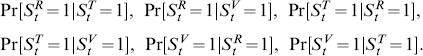



The collection of 60 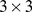 monthly overlap proportion matrices from the years 2005 to 2009 is constructed and summarized below.

**Figure 9:**
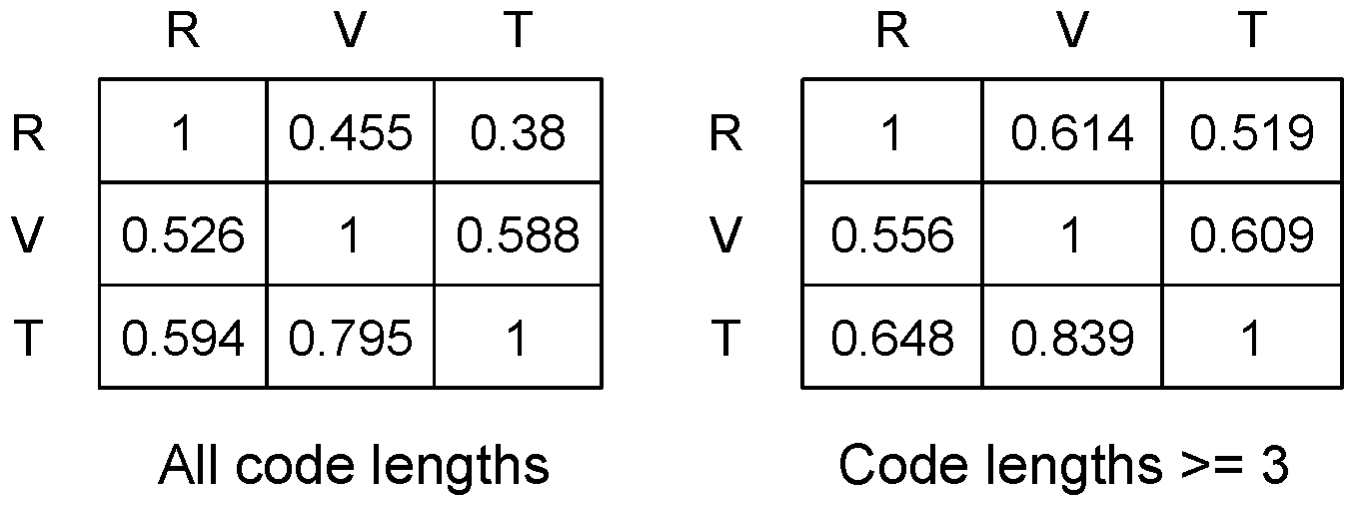
Overlap matrix of the month January crossing years 2005 to 2009.

These overlap matrices reflect an asymmetric binary relationship. This asymmetry is informative in the conditional sense. For instance, consider an averaged overlap matrix for the month January over the years 2005 through 2009, as given in Figure 9. When standing on an HFS-computed segment of the continuum of the return’s volatility, we can find that 45.5% of the time points are also coded with the trading volume’s volatility, and 38% with the transaction number’s volatility. In the reverse direction, 52.6% of the time points encoded with the trading volume’s volatility are also encoded with return’s volatility, while 59.4% of the time points encoded with the transaction number’s volatility are also encoded by the return’s volatility. These overlap percentages can rise significantly higher when we only consider code segments on 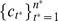 that are longer than or equal to three. It is interesting to see that the overlap proportion for trading volume relative to transaction number is 83.9%, while it is 60.9% in the reverse direction.

**Figure 10:**
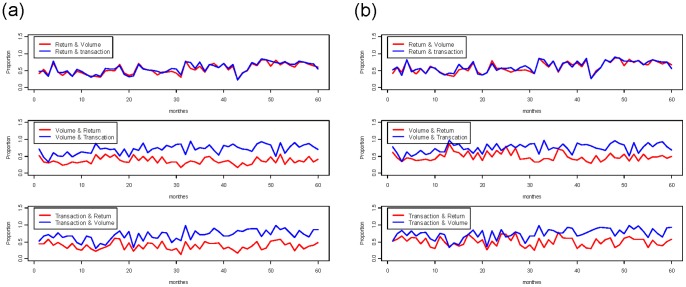
Comparing monthly overlap proportions among the volatilities of return, trading volume and transaction number of IBM stock from 2005 through 2009: a) for all code segments; b)for all code segments longer than or equal to 3.

In fact, we observe that the 60 overlapping matrices vary considerably from month-to-month as well as from year-to-year. The range is as wide as 20% to 100%. However, cases with around 20% overlap are not typical, while cases going beyond 80% are common, especially for the volatilities of transaction number and trading number in the years 2008 and 2009. These and other patterns are contained in the trajectories of overlap proportions shown in Figure 10. We further observe that both overlap relationships of the volatilities for trading volume and transaction number with respect to the return’s volatility are nearly equal throughout the five-year period. The two proportions are significantly higher than 50% for the entire year of 2009. A similar relationship in the reverse direction from return’s volatility to either trading volume’s volatility or transaction number’s volatility is not very different. The significant differences are found between the trajectory of the trading volume to the return and the trading volume to the transaction number. A similar difference is also found between the transaction number to the return and the transaction number to the trading volume. These significant differences confirm this fact: the potential of the trading volume and the transaction number to mutually stimulate each other is much higher than for either one of these two to stimulate the return.

In summary, these high overlap patterns strongly indicate that there is a closely intertwined relationship among the state of volatility for the three dimensions of stock dynamics. These close bonds among the three dimensions of a single stock explain the information revealed in Figure 6, in which we see that at least half of the synchrony is formed when the return’s volatility stimulates the volatilities of both the trading volume and the transaction number. Another third of the synchrony is formed in a reverse fashion: the return’s volatility is jointly stimulated by the volatilities of the trading volume and the transaction number.

## Results

Nowadays financial information is disseminated through the Internet at a rate of at least sub-seconds. Hence it is reasonable to imagine that all driving forces should act on stock dynamics at the same temporal scale. Indeed, traders and investors who make decisions using computer algorithms are capable of taking action at the sub-second or even the millisecond temporal scale. Consequently, the pertinent question arises: On what kind of temporal block should we carry out high-frequency data exploration for stock dynamics?

Since the logical requirement for exploring stock dynamics is, “We want to examine the trees, but also see the forest,” the significance of the above natural question rests in the balance between the global market pattern scale and the microstructure scale. The dynamic patterns we have computed and discussed up to this point should be taken as microstructure patterns at the daily or hourly scales, or even the minute scale. As for the global market pattern, we consider the contraction-expansion cycle, closely related to the business cycle in the overall financial market. We derive the contraction-expansion cycle as follows.

**Figure 11:**
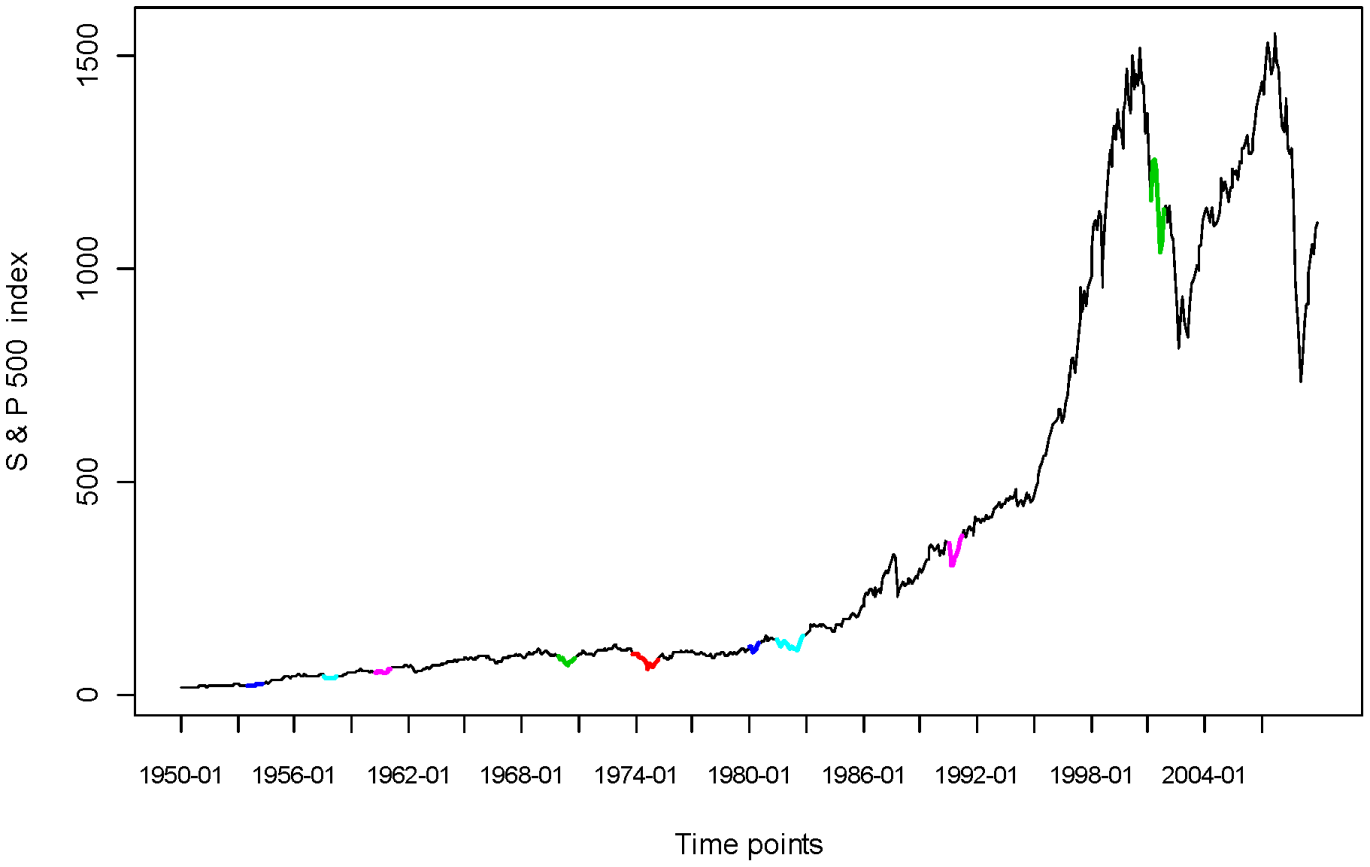
S&P500 Index from 1950 to 2009 marked with BCDC’s recession.

**Figure 12:**
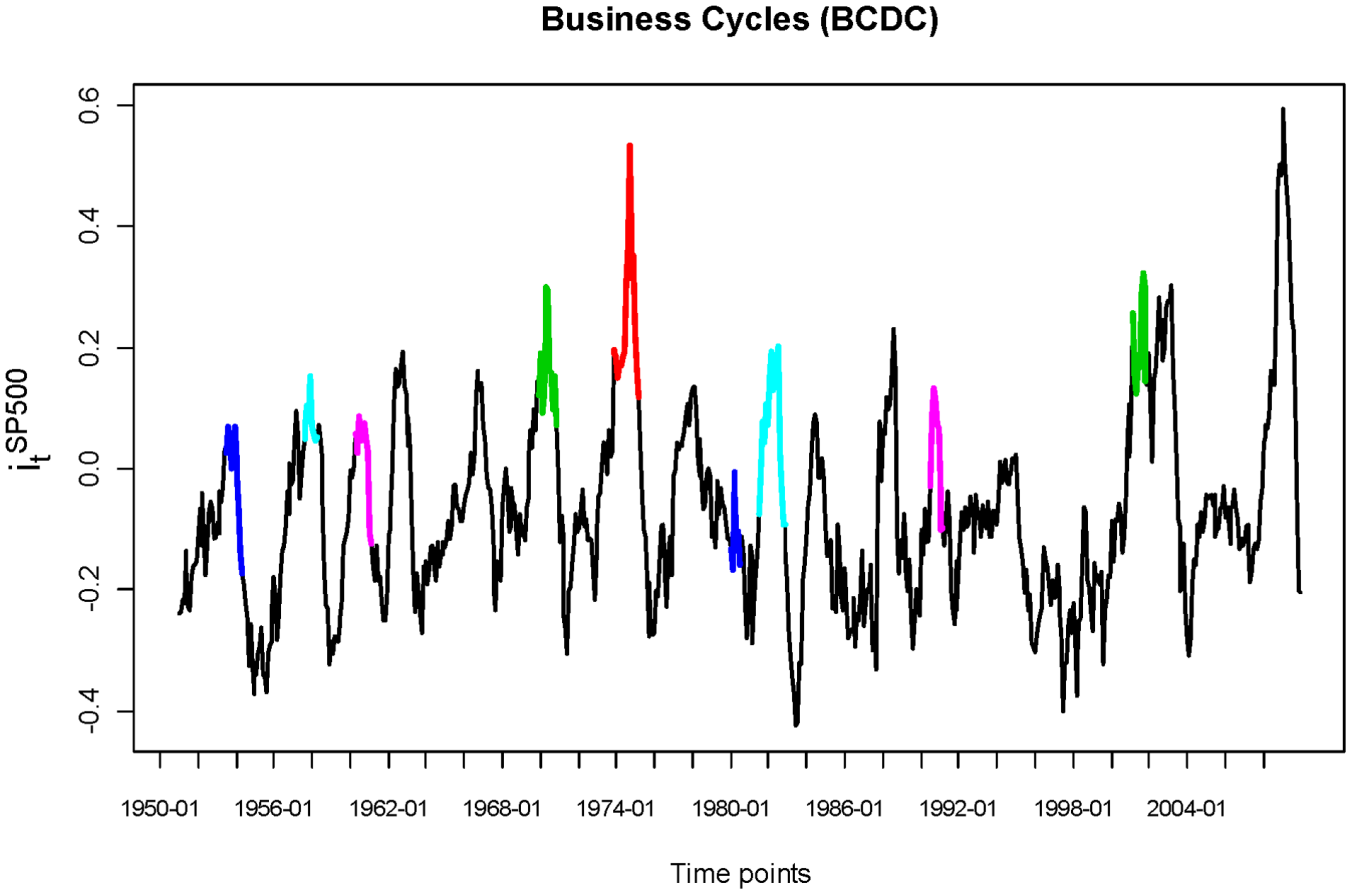
Monthly year-to-year S& P500.

In Figure 11, we see that the S&P500 index has a very steady trend, with two dips corresponding to two recessions: one in 2001 and the recent one starting from the end of 2007, as announced by the Business Cycle Dating Committee (BCDC) in the National Bureau of Economic Research (NBER). This S&P500 index trajectory does not reveal any “regularity” type of global pattern. However, if we transform the S&P500 index, say 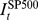, into a monthly year-to-year trajectory using the formula:
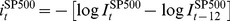
(5)with discrete 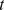 denoting the month in a year. The trajectory of 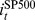 is observed to have some sort of cyclic regularity, as shown in Figure 12. It is essential to note that almost all recessions of the business cycle declared by the BCDC reside on the tips of recurrent spikes, due to the negativity of the transformed index. This pattern strongly and reliably indicates that every spike of 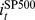 reveals a contraction period, and each valley an expansion period, in the stock market.

This regularity is brought out by the monthly data. Therefore a natural answer to the above question must be that a month is a suitable temporal block for exploring stock dynamics within high-frequency data. This is why all our analyses are done month-by-month throughout the 2005 to 2009 period.

**Figure 13:**
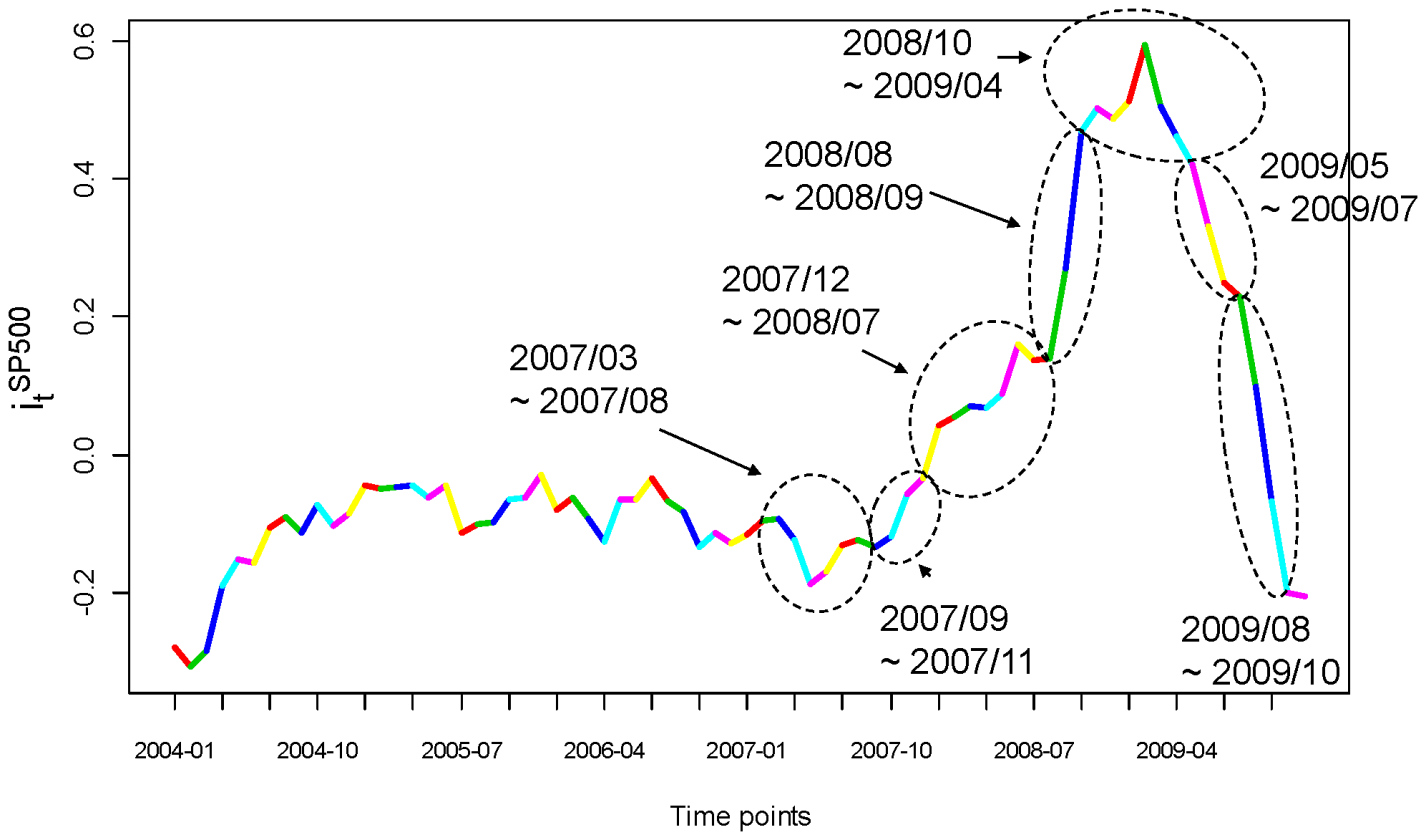
S&P500 Index (months of years) from 2004 to 2009.

**Figure 14:**
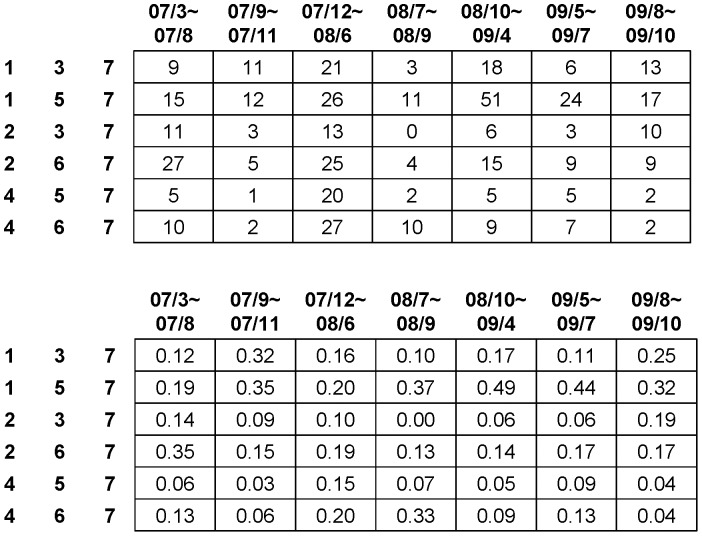
Frequency table of triple codes: 137, 157, 237, 267, 457 and 467 in different locality of contraction-expansion cycle.

In Figure 13, we further dissect the period from March of 2007 to October of 2009 into seven temporal segments. Each temporal segment bears significantly-different contraction and expansion global market patterns. We then look into the frequency of order-3 codewords leading to synchrony, as reported in Figure 14. The most vivid dynamic pattern is the nearly 50% synchronization of volatility during the period from August 2008 to April 2009, coinciding with the deepest point of the most recent recession. This is denoted by codeword 157, since the return volatility stimulates transaction’s volatility and then trading number’s volatility. A similar pattern is also seen in the next temporal segment, from May 2009 to July 2009, with slightly less synchronization–but still an equally significant level–when combining the two codewords 137 and 157. In contrast, during the beginning of the recession from December 2007 to June 2008, the occurrences of synchrony are much more frequent than in all other temporal segments. In the same temporal segment and in the one that follows from July 2008 to September 2009, we see the frequency of codeword 467 is unusually high when compared with the rest of the temporal segments. This clearly indicates that trading strategies may have been implemented to create volatility of the transaction number in order to bring out the trading volume’s volatility and subsequently to generate the return’s volatility. The discovery of these non-stationary dynamic patterns confirms that a month is the right temporal block.

## Discussion

In this paper we employ the HFS algorithm to encode a single stock’s dynamics into a compressed digital code sequence of base 8. This digital code sequence facilitates many realistic dynamic patterns that characterize the stock’s underlying mechanism, which reveals fundamentally different characteristics from what are prescribed by current popular theories in finance. The major findings are: 1) The primary driving force of stock dynamics is the return’s volatility, which is responsible for stimulating volatilities in the trading volume and the transaction number dimensions; 2) The secondary driving force is the combined, but not individual, volatilities of the trading volume and the transaction number, which is capable of stimulating the return’s volatility; 3) A single stock’s dynamic patterns can vary as the market goes into and out of the contraction-expansion cycle. These findings should broaden our understanding of a single stock’s dynamics. We once again emphasize the necessity of embedding all computed dynamic patterns onto the market’s global trends in order to extract and confirm their realistic essence. The contraction-expansion cycle is one especially important global trend, since it depicts the non-stationarity in the financial market that influences as well as coordinates the flow of all involved dynamic patterns underlying every single stock’s dynamics.

In this paper we also demonstrate that the computational approach using the HFS algorithm is rather promising for exploring stock dynamics in depth. Our computed dynamic patterns are very credible, since they indicate a very high overlap proportion between the involved volatilities of the three stock dimensions. The overlap proportion matrix also provides a new way for evaluating the association between two time series. This association measurement should be more proper for evaluating dynamic patterns computed from high-frequency time series than that based on correlation.

In our future research, we will study how to construct a stock dynamics model that is consistent with the explored and computed dynamic patterns. We hope this research can ideally lead to the construction of a platform for accommodating a mechanism for pricing, like the Black-Scholes model [15].

At the end, we briefly discuss the potential impact of high-frequency trading via computer algorithms on stock dynamics. The algorithmic trading is very far away from what is done on the floor or what is screen-based [16]. It can place many orders, and at the same time cancel many orders, to thus jump ahead of all traders using traditional instruments. The ask-bid spread can be so small that we start to wonder how much truth still resides in the old understandings in finance, such as: 1) Trading is generated due to asymmetric information received by traders; 2) The size of trades reflects the extent of disagreement among traders about a security’s value.

It is very likely that there is still some truth left nowadays, given that a handful of high-frequency trading firms accounted for an estimated 70% of the overall trading volume on the U.S. equities markets in 2009 [16]. That is, several computer algorithms together are responsible for the whole stock dynamic, to an overwhelmingly large degree. Its fundamental setting has shifted to the interacting relationships among a handful of computer algorithms. Hence we intuitively suspect that stock dynamics may have undergone many drastic changes during the era of computer algorithmic trading.
